# 

**Published:** 1868-12-15

**Authors:** 


Vol. XXV. Number 24.

### THE CHICAGO MEDICAL JOURNAL.

*PUBLISHED SEMI-MONTHLY.*

J. Adams Allen, M.D., LL.D.

EDITOR.

Walter Hay, A.M., M.D.

ASSOCIATE EDITOR.

DECEMBER 15, 1868.

CHICAGO:

Church, Goodman and Donnelley, Steam Book and Job Printers,

108 and 110 Dearborn Street.

